# Effectiveness of same-day antiretroviral therapy initiation in retention outcomes among people living with human immunodeficiency virus in Ethiopia: empirical evidence

**DOI:** 10.1186/s12889-020-09887-9

**Published:** 2020-11-26

**Authors:** Ismael Ahmed, Meaza Demissie, Alemayehu Worku, Salem Gugsa, Yemane Berhane

**Affiliations:** 1 University of Gondar, Gondar, Ethiopia; 2 Addis Continental Institute of Public Health, Addis Ababa, Ethiopia; 3 Department of Preventive Medicine, School of Public Health, College of Health Sciences, Addis Ababa University, Addis Ababa, Ethiopia; 4 Department of Global Health, University of Washington, Seattle, WA USA

**Keywords:** Same-day antiretroviral therapy, Rapid ART, Retention, Attrition, Test and treat, Africa, Ethiopia

## Abstract

**Background:**

In August 2016, Ethiopia endorsed a universal “test and treat” strategy for people living with human immunodeficiency virus (PLHIV) based on World Health Organization recommendation. However, there is limited evidence on the routine application of the same-day “test and treat” recommendation in low-income settings. This study assessed the effect of same-day treatment initiation on individual-level retention at 6- and 12-months follow-up.

**Methods:**

A multicenter facility-based retrospective cohort study was conducted to compare retention-in-care between PLHIV who started antiretroviral therapy (ART) on the same-day and those started ART > 7 days following HIV diagnoses. Participants were at least 15 years-old and were newly diagnosed and started on ART between October 2016 and July 2018 in 11 health facilities in the Amhara region of Ethiopia. Multivariable logistic regression controlling for potential confounders and Kaplan-Meier survival analysis were used to assess differences in outcomes between the groups.

**Results:**

In total, 433 PLHIV started ART on the same-day of diagnosis and 555 PLHIV who started ART > 7 days after HIV diagnosis were included in the study. At 6-months, 82.0% (355) in the same-day group vs 89.4% (496) in the > 7 days group were retained-in-care (absolute risk difference (RD) = 7.4%; 95% confidence interval (CI): 2.9–11.8%). At 12-months, 75.8% (328) in the same-day group vs 82.0% (455) in the > 7 days group were retained-in-care (absolute RD = 6.2%; 95% CI: 1.1, 11.4%). The major drop in retention was in the first 30 days following ART initiation among same-day group. After adjusting for baseline and non-baseline covariates, the same-day group was less likely to be retained-in-care at 6- and 12-months (adjusted risk ratio (RR) = 0.89; 95% CI: 0.87, 0.90 and adjusted RR = 0.86; 95% CI: 0.83, 0.89, respectively).

**Conclusions:**

Reduced retention-in-care can threaten the benefit of the same-day “test and treat” policy. The policy needs to be implemented cautiously with greater emphasis on assessment and preparation of PLHIV for ART to ensure treatment readiness before starting them on same-day ART and close monitoring of patients during early follow-up periods.

**Supplementary Information:**

The online version contains supplementary material available at 10.1186/s12889-020-09887-9.

## Background

Despite increased access to and utilization of human immunodeficiency virus (HIV) testing and treatment services, attrition after being initiated on antiretroviral therapy (ART) has been a prevailing challenge for ART programs in low-income settings (LIS). Various systematic reviews conducted on ART programs in low- and middle-income countries [1, 2] and specifically in Ethiopia [3, 4] show high attrition from ART programs due to death or loss to follow-up (LTFU). Patients started on ART with advanced World Health Organization (WHO) clinical stage (III/IV) or bed ridden/seriously ill functional status [5–8] or lower CD4 cell count [8–10] had increased risk of attrition. This was mainly linked with delayed treatment initiation [5–7] that resulted from restricted initiation criteria following the earlier WHO guidance [11]. Increased mortality was also observed during the first 6-months of ART initiation [5–7, 9] which is likely related to the late presentation of patients for ART initiation [9].

Since 2013, evidence have emerged showing that early initiation of ART results in better clinical outcomes for people living with HIV (PLHIV) compared to delayed treatment in LIS [12–14]. Accordingly, in 2016, the WHO released a recommendation to provide universal “test and treat” services for PLHIV, regardless of their CD4 cell count or WHO clinical stage, in order to improve clinical outcomes. In addition, this universal eligibility strategy aimed to increase access to antiretroviral (ARV) drugs for treating and preventing HIV, and thereby achieve the “goal of ending the HIV epidemic as a major public health threat by 2030” [15].

Ethiopia adopted “immediate” ART initiation in August 2016 [16], and revised the national consolidated ART guideline again in August 2018 with more clear guidance on how soon to start treatment, including same-day ART initiation [17] following a WHO update that supports rapid ART initiation (within 1 week of HIV diagnosis) in July 2017 [18]. During this transitional time, some clinicians and HIV program managers were reluctant to initiate individuals on ART on the same-day of HIV diagnosis due to fears of poor adherence and attrition.

As same-day “test and treat” is a new initiative in LIS, there is a dearth of evidence regarding the effectiveness of ART initiation on the day of HIV diagnosis. Findings from the available Randomized Controlled Trails (RCTs) conducted on same-day ART initiation in Haiti [19] and Lesotho [20] are hard to extrapolate to ART clinics that lack basic resources for routine service delivery. Recent observational studies that evaluated the national scale-up of “test and treat” in South Africa [21] and Haiti [22] highlighted that although a higher proportion of PLHIV were started on ART on the same-day of diagnosis, those started on ART on same-day of HIV diagnosis were less likely to be retained-in-care 6-months post-initiation of treatment in the initial year of implementing “test and treat”. A similar program evaluation in Nigeria reported that one-third of PLHIV who initiated on ART within 2 weeks of HIV diagnosis were LTFU, of whom more than half were lost within the first 30-days [23]. On the other hand, emerging results from studies in Africa have shown that certain group of individuals such as PLHIV with younger age [21, 23, 24], higher WHO clinical stage, higher baseline CD4 cell count, and physically affected functional status [23] have increased risk of LTFU after rapid ART initiation. Overall, despite the success of increased proportions of PLHIV enrolling for ART on the same-day of diagnosis [21, 22, 25], retaining PLHIV on ART in routine program settings remains a challenge. This may be due to initiation of ART without adequate preparation of PLHIV and assessment of their fully informed readiness for lifelong treatment [17]. In this study we sought to describe 6- and 12-months retention-in-care in PLHIV who were initiated on ART on the same-day of HIV diagnosis vs > 7 days after HIV diagnosis, and assess the differences in retention-in-care before and after adjustment for observed differences in the two groups.

## Methods

### Study design

We used a multicenter retrospective cohort study design to compare the clinical outcomes of individuals who started ART on the day of HIV diagnosis (exposed) versus initiation after 7 days (non-exposed). To do this, routine clinical service delivery data were abstracted from facility-based medical records.

### Study setting

The study was conducted at public health facilities in Bahir Dar and Gondar, towns in the Amhara region of northwest Ethiopia. The region shares more than 30% of the national HIV burden [26]. Out of 16 ART service providing public facilities in the two towns, 11 health facilities (eight health centers and three hospitals) that had > 20 PLHIV on ART by the end of December 2018 were included in the study. We excluded one newly established health center that provided ART service mainly for transferred-in (TI) persons from other health facilities during the study period. The participating facilities provide comprehensive HIV services such as HIV prevention, testing, care and treatment and laboratory services. They also have access to centralized viral load testing laboratories in their respective towns. Following ART initiation, viral load testing is done to routinely monitor virologic suppression at 6-months, at 12-months and then every 12-months thereafter. HIV services are provided by multidisciplinary teams composed of physicians, health officers, nurses, pharmacy technicians, laboratory technicians, adherence case managers, adherence supporters, and data personnel. The ART initiation and follow-up services are mainly provided by nurses who underwent ART training based on the national consolidated ART training manual. According to the latest national ART guideline, PLHIV are immediately linked to an ART clinic for a confirmatory test, counseling, adherence preparation and rapid ART initiation - including same-day ART for persons who are ready to start ART at the first clinical visit and have no sign of opportunistic infections (OI) (e.g. Tuberculosis (TB) and cryptococcal meningitis) that would result in delayed ART initiation [17]. The guideline recommends baseline laboratory tests such as CD4 cell count, CBC, ALT, and creatinine, if available. PLHIV who have no sign of OI but deferred rapid ART initiation were counseled and given an appointment to return a week following their HIV diagnosis for initiation. This scheme – counseling and making an appointment for a person to start ART a week later – was similar to ART implementation prior to the “test and treat” era [27]. After initiation, PLHIV were asked to return to ART clinic for monitoring at ½-, 1-, 3-, 4-, and 6-months, and every 3 months thereafter irrespective of time of ART initiation [17].

### Participants

This study included two groups of PLHIV who were newly diagnosed and started on ART at the study sites; those who were started on the same-day as HIV diagnosis and those who were started > 7 days after the initial diagnosis. Eligible PLHIV were ≥ 15 years-old and newly initiated on ART between 20 October 2016 (the time when study sites started same-day ART initiation) and 18 July 2018. Records of ART clients who were initiated on ART 1–7 days after diagnosis, aged < 15 years old, pregnant, TI from another health facility, and transferred-out (TO) to another health facility within 12-months of ART initiation were excluded from the study. In addition, persons whose rapid ART initiation was delayed for clinical reasons (e.g. TB and cryptococcal meningitis) [17] were excluded from the study.

### Variables and measurement

The primary outcome of this study was retention-in-care at 6- and 12-months, which was defined as PLHIV known to be alive and receiving ART at the end of a follow-up periods [17, 28]. LTFU and death at 6- and 12-months were secondary outcomes of the study. Individuals who did not refill their ART for a period of one or more month after their last refill appointment date and were not yet classified as having died or TO were labeled as LTFU. However, individuals who discontinued treatment but returned to care before 6- and 12-months post-ART initiation were considered as retained at 6- and 12-months, respectively. Death was defined as all-cause death for clients whose death was recoded on the patient’s medical record or ART register.

Independent factors considered in the analysis include biological, sociodemographic and clinical factors. Biological factors included CD4 cell count and body mass index (BMI). Sociodemographic factors included age, sex, marital status, education, religion, residence, partner’s HIV status and disclosure of HIV status. WHO clinical stage, functional status, type of ARV regimen initiated and taking cotrimoxazole preventive therapy (CPT) and isoniazid preventive therapy (IPT) were used to measure clinical condition. All variables were measured at baseline except for: partner’s HIV status which was measured by the 6-month time point and use of CPT and IPT by the 6- and 12-month time point. Type of ARV regimen was measured at ART initiation and if regimen was switched.

Data were abstracted from the patients’ intake and follow-up forms completed during enrollment and routine follow-up in the ART clinics. The data were captured using the Open Data Kit electronic system to ensure efficient and validated data-entry, using pre-programmed options for a majority of the data entered [29]. Data of eligible persons were abstracted from medical records by 11 experienced ART prescriber nurses working at the ART clinics. Data quality assurance and on-site support for data abstraction was provided by the study team.

### Study size and sampling method

**Fig. 1:**
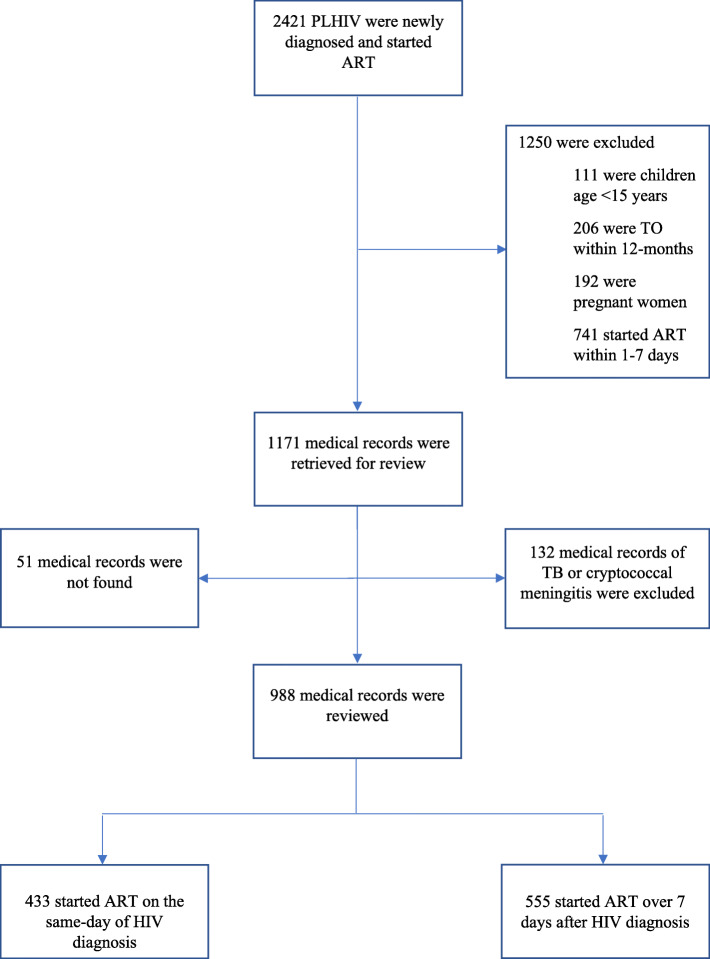
Summary of number of participants and reasons for enrollment in the study

The sample size was determined using StatCalc for cohort studies using Epi Info™ version 7 (developed by Centers for Disease Control and Prevention). We estimated the proportion of retention among PLHIV who started ART on the same-day of diagnosis and > 7 days after diagnosis based on a study from South Africa [30]. Accordingly, P_1_ of 81% (proportion of retention at 10-months of ART among persons in the same-day group), P_2_ of 64% (proportion of retention at 10-months of ART among individuals in the control group), a 1:1 proportion, α of 0.05, and 80% power was used to calculate sample size. We used a design effect of 1.5 to account for the effect of clustering in this multicenter design and increased the required sample size by 15% allowance for anticipated limitations with regard to missing medical record data in Ethiopia [6]. Based on this, we estimated that we would need to enroll a minimum of 420 participants in both groups. However, in order to increase the power of the study, all eligible individuals who were newly diagnosed and started on ART between 20 October 2016 and 18 July 2018 were included in the study, making the total sample size 988 (433 same-day and 555 after 7 days) (Fig. 1).

### Statistical analysis

Data were exported to Stata version 13.0 (StataCorp., College Station, TX) for further cleaning and data analysis. Baseline characteristics of study participants were summarized using descriptive statistics. Proportions of persons retained-in-care, LTFU and died at 6- and 12-months after enrollment were compared with absolute risk difference (RD) between the two groups using a chi-square test. Time-to-event methods (Kaplan–Meier plots and the log-rank test) were used to compare the cumulative probability of retention in comparison study groups. Person-time accrued from ART initiation date to the earliest of LTFU or death was estimated to compare incidence of attrition between the two groups.

We determined the missingness of the variables to be “missing completely at random” based on our knowledge of the HIV program and Little’s chi-square test, and employed complete case analysis [31]. However, during analysis we excluded baseline CD4 cell count from the analysis due to a high proportion of missing data which was significantly different between the two comparison groups.

We compared the proportions of individuals achieving the primary (retention-in-care) and secondary (LTFU and death) outcomes at the end of 6- and 12-months using unadjusted and adjusted risk ratio (RR) with 95% confidence interval (CI) for each study group.

We checked the balance between the two comparison groups using a chi-square test. To estimate the adjusted effects, we first balanced the two groups by estimating the propensity score using a logistic regression model [32, 33] to adjust for the baseline covariates such as age, sex, marital status, education, place of residence, BMI, WHO clinical stage, functional status and OI at enrollment. We used doubly-robust multivariable logistic regression model using backward stepwise variable selection that included both the propensity score and the baseline covariates to ensure sufficient covariate balance [32, 33]. We also considered other non-baseline covariates in the model such as CPT, IPT, type of ARV regimen initiated, disclosure of HIV status and partner’s HIV status. We accounted for the effect of clustering at town level using cluster-robust standard error [34, 35]. We explored all possible interaction terms between the main effect variables. The final model that included the propensity score and all significant variables (*p* < 0.05) was tested for goodness-of-fit using the Hosmer-Lemeshow test supplemented by c-statistics for the area under the receiver operator characteristic curve [36]. Adjusted RR with 95% CI was computed after running the final logit model [37].

We conducted a sensitivity analysis for the primary outcome by excluding individuals with WHO clinical stages III or IV to observe the effects of treatment in persons who were less likely to have advanced disease events [18]. The sensitivity analysis excluded 219 study participants (39 same-day and 180 > 7 days starters) with WHO stage III or IV.

## Result

### Characteristics of study participants

Of the 2421 people who were newly diagnosed and started on ART at the participating health facilities between 20, October 2016 and 18, July 2018, a total of 433 same-day starters and 555 who started > 7 days after the initial diagnosis were eligible to be included in our study (Fig. 1).

More than half (52.6%, *n* = 520) of study participants were women. The overall median age at ART initiation was 33 years (IQR 27, 40 years). The majority were married (42.3%, *n* = 396), resided in town (80.8%, *n* = 795), presented with WHO clinical stage I (57.1%, *n* = 564), had no OI at enrollment (81.9%, *n* = 809), and had a median BMI of 20 (IQR 18, 22). Of the total 988 study participants, only 57.8% (*n* = 571) had documented baseline CD4 cell count result with significant missing data difference between the two groups (264 (61.0%) in the same-day group and 153 (27.6%) in the > 7 days group; *p < .001)*.

Baseline and follow-up socio-demographic and clinical characteristics of participants in each study group are shown in Tables 1 and 2. Persons who were initiated on ART on same-day of and > 7 days after diagnosis were similar in sex, median age, marital status, religion, partner’s HIV status, and disclosure. However, in the same-day group, there were higher proportions of individuals with WHO clinical stage I (74.6% (*n* = 323) vs 43.4% (*n* = 241)) and no OI (91.5% (*n* = 396) vs 74.4% (*n* = 413)) at enrollment compared to the > 7 days group. In the > 7 days group, higher proportions of study participants were taking CPT by 6-months (67.2% (*n* = 373) vs 28.6% (*n* = 124)) and 12-months (60.4% (*n* = 335) vs 23.8% (*n* = 103)) time point compared to the same-day group. On the other hand, compared to PLHIV who started ART > 7 days of HIV diagnosis, there were higher proportions of individuals who were taking IPT within the first 6-months (63.1% (*n* = 273) vs 38.4% (*n* = 213)) and 12-months (62.1% (*n* = 269) vs 46.7% (*n* = 259)) of initiating same-day ART. While both groups of study participants started with a Non-nucleoside Reverse Transcriptase Inhibitors (NNRTI)-based regimens, almost all (99.5%, *n* = 431) of the same-day group started on a fixed-dose combination (FDC) of Tenofovir (TDF) + Lamivudine (3TC) + Efavirenz (EFV) compared to 96.0% (*n* = 533) of the > 7 days group. Due to side effects or drug toxicity, 11 people in the > 7 days group had their regimen changed to a different first-line ART in the 12-months following ART initiation. In addition, one person’s ART in each group was changed to second-line ART within 12-months of ART initiation.

**Table 1:** Sociodemographic characteristics of study participants by group (October 20, 2016 to July 18, 2018)

Characteristics	Same-day group n (%)	> 7 days group n (%)
Sex (*N* = 988)		
Male	190 (43.9)	278 (50.1)
Female	243 (56.1)	277 (49.9)
Age in years (*N* = 988) – Median (IQR)	32 (27.0–39.0)	34 (28.0–40.0)
Educational status (*N* = 924)^†^		
No Education	107 (26.4)	154 (29.7)
Primary	121 (29.9)	170 (32.8)
Secondary	130 (32.1)	122 (23.5)
Tertiary	47 (11.6)	73 (14.1)
Marital status (*N* = 936)		
Never married	97 (23.3)	124 (23.9)
Married	171 (41.0)	225 (43.4)
Divorced/separated	126 (30.2)	147 (28.3)
Widow/er	23 (5.5)	23 (4.4)
Religion (*N* = 979)		
Orthodox	389 (90.9)	507 (92.0)
Protestant	3 (0.7)	3 (0.5)
Muslim	36 (8.4)	41 (7.4)
Place of residence (*N* = 984)^†^		
Within town	368 (85.2)	427 (77.4)
Out of town	64 (14.8)	125 (22.6)
Disclosure of HIV+ status (*N* = 728)*		
Disclosed	291 (85.3)	329 (84.2)
Not disclosed	50 (14.7)	61 (15.8)
HIV status of partner (*N* = 862)*		
HIV negative	54 (14.2)	68 (14.1)
HIV positive	99 (26.0)	114 (23.7)
Unknown	46 (12.1)	58 (12.1)
No partner	182 (47.8)	241 (50.1)
Functional status (*N* = 986)^†^		
Working	418 (96.5)	479 (86.6)
Ambulatory	14 (3.2)	52 (9.4)
Bed ridden	1 (0.2)	22 (4.0)

†Variables significantly different between the two groups at *P < 0.05* level, based upon χ2 test for equality of proportions. *In subsequent follow-up visits post-treatment initiation

*HIV* human immunodeficiency virus, *IQR* interquartile range

**Table 2:** Clinical characteristics of study participants by group (October 20, 2016 to July 18, 2018)

Characteristics	Same-day group n (%)	> 7 days group n (%)
BMI (*N* = 987)—Median (IQR)^†^	20.1 (18.3–22.3)	19.6 (17.5–22.0)
CD4 cell count/mm^3^ (*N* = 571)^†^		
< 200	58 (34.3)	218 (54.2)
200–349	31 (18.3)	86 (21.4)
≥ 350	80 (47.3)	98 (24.4)
WHO clinical stage (*N* = 988)^†^		
Stage I	323 (74.6)	241 (43.4)
Stage II	71 (16.4)	134 (24.1)
Stage III	35 (8.1)	141 (25.4)
Stage IV	4 (0.9)	39 (7.0)
OI at enrollment (*N* = 988)^†^		
Yes	37 (8.6)	142 (25.6)
No	396 (91.5)	413 (74.4)
CPT within 6-months (*N* = 988)^†^ *		
Yes	124 (28.6)	373 (67.2)
No	30 (6.9)	17 (3.1)
Not eligible	276 (64.4)	165 (29.7)
CPT within 12-months (*N* = 988)^†^ **		
Yes	103 (23.8)	335 (60.4)
No	43 (9.9)	44 (7.9)
Not eligible	287 (66.3)	176 (31.7)
IPT within 6-months (*N* = 988)^†^ *		
Yes	273 (63.1)	213 (38.4)
No	142 (32.8)	275 (49.6)
Not eligible	18 (4.2)	67 (12.1)
IPT within 12-months (*N* = 988)^†^ **		
Yes	269 (62.1)	259 (46.7)
No	139 (32.1)	229 (41.3)
Not eligible	25 (5.8)	67 (12.1)
ARV regimen started (*N* = 988)^†^		
TDF + 3TC + EFV (FDC)	431 (99.5)	533 (96.0)
AZT + 3TC + EFV	1 (0.2)	10 (1.8)
Others	1 (0.2)	12 (2.2)

†Variables significantly different between the two groups at *P < 0.05* level, based upon χ2 test for equality of proportions. *Within 6-months of ART initiation. ** Within12-months of ART initiation

*ARV* antiretroviral, *AZT* Zidovudine, *BMI* body mass index, *CPT* cotrimoxazole preventive treatment, *FDC* fixed dose combination, *EFV* Efavirenz, *IPT* isoniazid preventive therapy, *IQR* interquartile range, *3TC* Lamivudine, *OI* opportunistic infection, *TDF* Tenofovir, *WHO* World Health Organization

### Retention-in-care at 6- and 12-months of ART follow-up

Retention at 6-months was 82.0% (*n* = 355) in the same-day group and 89.4% (*n* = 496) in the > 7 days group, indicating an absolute RD of 7.4% (95% CI: 2.9, 11.8%; *p < .001*). Similarly, 12-months retention was lower in the same-day group (75.8%, *n* = 328) compared to the > 7 days group (82.0%, *n* = 455) with an absolute RD of 6.2% (95% CI: 1.1, 11.4%; *p = .02*) (Table 3).

**Table 3:** Outcomes of ART initiation at 6- and 12-months ART follow-up by group

Outcomes	No. (%) of participants		Absolute RD, % (95% CI)	***p***-value
	Same-day (***n*** = 433)	> 7 days (***n*** = 555)		
**Primary outcomes**				
Retention at 6-months	355 (82.0)	496 (89.4)	7.4 (2.9, 11.8)	< 0.001
Retention at 12-months	328 (75.8)	455 (82.0)	6.2 (1.1, 11.4)	0.02
**Secondary outcomes**				
LTFU at 6-months	63 (14.5)	33 (5.9)	8.6 (4.7, 12.5)	< 0.001
LTFU at 12-months	87 (20.1)	62 (11.2)	8.9 (4.3,13.5)	< 0.001
Death at 6-months	15 (3.5)	26 (4.7)	1.2 (−1.2, 3.7)	0.34
Death at 12-months	18 (4.1)	38 (6.8)	2.7 (−0.1, 5.5)	0.07

*LTFU* loss to follow-up, *RD* risk difference

**Fig. 2:**
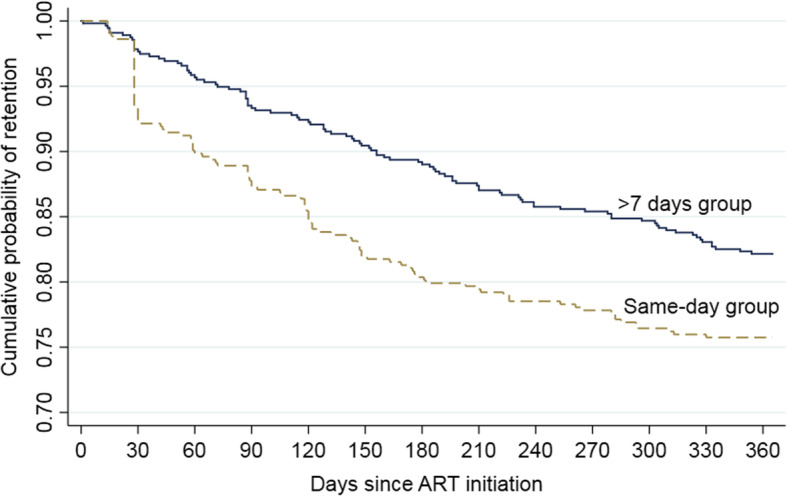
Survival plots based on Kaplan–Meier estimates comparing retention since ART initiation by study groups

Figure 2 demonstrates the cumulative probability of retention in each study group over the 365 days following treatment initiation. It shows that the probability of retention was significantly lower in same-day group, (*P = .008*, based on the log-rank test; corresponding χ2 = 7.14). The major drop in retention probability was in the first 30-days among the same-day group. In other words, attrition was higher in the same-day group (8.8/person-months) compared to the > 7 days group (6.0/person-months) with incidence rate ratio of 1.5 (95% CI: 1.10, 2.0; *p = .006*) during the 12-months follow-up time.

Compared to the > 7 days group, the unadjusted and adjusted RR of 6-months retention for same-day group were 0.92 (95% CI: 0.87, 0.97; *p < 0.001*) and 0.89 (95% CI: 0.87, 0.90; *p < .001*), respectively. Similarly, the unadjusted RR of being retained at the end of 12-months of follow-up for the same-day group was 0.92 (95% CI: 0.86, 0.99; *p = .02*) compared to the > 7 days group; the adjusted RR for this comparison was 0.86 (95% CI: 0.83, 0.89; *p = <.00 l*) (Table 4).

**Table 4:** Unadjusted and adjusted RR of study outcomes for same-day ART group

Outcomes	Unadjusted		Adjusted**	
	RR*	95% CI	RR*	95% CI
Retention at 6-months^a^	0.92	(0.87, 0.97)	0.89	(0.87, 0.90)
Retention at 12-months^b^	0.92	(0.86, 0.99)	0.86	(0.83, 0.89)
LTFU at 6-months^c^	2.45	(1.64, 3.66)	2.92	(1.57, 5.42)
LTFU at 12-months^d^	1.80	(1.33, 2.43)	2.11	(1.70, 2.61)
Mortality at 6-months^e^	0.74	(0.40, 1.38)	1.26	(0.93, 1.71)
Mortality at 12-months^f^	0.61	(0.35, 1.05)	0.94	(0.63, 1.40)

*LTFU* Loss to follow-up, *RR* risk ratio

*Reference group = persons initiated on ART > 7 days after HIV diagnosis

**Multivariable logistic regression model included the propensity score and other covariates such as:

^a^age, BMI, CPT, IPT, baseline OI, baseline functional status and partner’s HIV status

^b^age, gender, BMI, IPT, baseline OI, baseline functional status, baseline WHO clinical stage, and partner’s HIV status

^c^age, gender, education status, CPT, IPT, baseline OI and partner’s HIV status

^d^age, gender, IPT, baseline OI, baseline WHO clinical stage and partner’s HIV status

^e^age, educational status, BMI, baseline OI and baseline WHO clinical stage

^f^age, educational status, BMI, CPT, IPT and baseline functional status

### LTFU and mortality at the end of 6- and 12-months of ART follow-up

As reported in Table 3, 6-months LTFU was significantly higher in the same-day group (14.5%) compared to the > 7 days group (5.9%) with an absolute RD of 8.6% (95% CI: 4.7, 12.5%; *p < .001*). At 12-months, similarly LTFU was significantly higher in the same-day (20.1%) compared to those initiated > 7 days (11.2%) with an absolute RD of 8.9% (95% CI: 4.3, 13.5%; *p < .001*). Furthermore, compared to > 7 days group, the adjusted RR of 6-months LTFU for same-day group was 2.92 (95% CI: 1.57, 5.42; *p = <.001*). The adjusted RR of 12-months LTFU for the same-day group was 2.11 (95% CI: 1.70, 2.61; *p = <.001*), compared to the > 7 days group. There was no statistically significant differences or risk in mortality at 6- and 12-months between the two groups (Table 4).

### Sensitivity analysis

In a sensitivity analysis that included only study participants with WHO clinical stage I and II, retention was significantly lower in the same-day group (Additional file 1). Retention at 6-months was 82.2% (*n* = 324) in same-day group and 92.3% (*n* = 345) in > 7 days group with an absolute RD of 9.8% (95% CI: 5.1, 14.4%; *p < .001*). Similarly, at the end of 12-months follow-up time, retention was lower in the same-day group (76.1%, *n* = 300) compared to the > 7 days group (85.1%, *n* = 319) with an absolute RD of 8.9% (95% CI: 3.4, 14.5%; *p = .002*). The adjusted RR of 6-months retention for the same-day group was 0.86 (95% CI 0.81, 0.90; *p < .001*) compared to the > 7 days group. Similarly, the adjusted RR of 12-months retention for the same-day group was 0.86 (95% CI: 0.83, 0.89; *p < .001)* compared to the > 7 days group.

## Discussion

Our findings demonstrated that individuals who started ART on the same-day of HIV diagnosis had lower retention at 6-months (82.0% vs 89.4%) and 12-months (75.8% vs 82.0%) of ART follow-up compared to those who started ART > 7 days after their HIV diagnosis. The cumulative probability of retention was significantly lower in the same-day group whereby the major drop in retention happened in the first 30-days of same-day ART initiation. Overall, attrition in the same-day group (8.8/person-month) was greater than in those who initiated ART 1 week later (6.0/person-month).

The evidence in this study was generated using data from the routine clinical care settings in Ethiopia. We applied strict eligibility criteria to include study participants and involved multiple sites with a fairly large sample size [38]. We also used propensity score matching to balance the two comparison groups [33, 34]. So, we believe this study provides a fairly valid information on the subject matter under routine program conditions.

Even though two earlier RCTs demonstrated the efficacy of same-day ART initiation in improving retention of PLHIV at 12-months [19, 30], empirical observational studies raise concerns about whether the benefits measured in RCTs fully translate into real-world settings [21, 22]. Our study, reflecting real-world clinical care settings, shows lower retention among individuals who start ART on the day of HIV diagnosis compared to those starting > 7 days following diagnosis. Given the controlled and well-resourced nature of most RCTs, their generalizability to ordinary ART clinics is imperfect. For instance, in RCT conducted in Haiti [19] at a non-governmental urban clinic which was differently equipped in terms of infrastructure and staffing (physician evaluation and social worker counseling), participants were limited to PLHIV with disease in WHO clinical stages I or II and CD4 count < 500 cells/mm^3^ [19]. Additionally, participants were supported by accelerated counseling protocols, intensified early visit schedule, and transportation subsidy which are support systems that are rare during routine public health service provision [19]. In support of this argument, an observational study from the same setting (Haiti) found results that fell short of the benefit observed in the RCT [22].

Our study also demonstrated that there was significant attrition in the first month following same-day ART initiation. Similarly, more than half of LTFU PLHIV who initiated under “test and treat” strategy in Nigeria were LTFU within the first 30-days [23]. A high proportion of early program LTFU was also seen in South Africa where 35% of same-day initiators who were LTFU did not return to the ART program after the first initiation visit [21]. The Haiti observational study also showed that 20% of individuals who were initiated on same-day ART failed to return for ART refill after the initial ART dispensation [22]. These evidence show that there could be individuals who were not ready or not eligible for immediate ART initiation [15] but started on rapid ART without adequate assessment and support to address risk factors for attrition including barriers to treatment adherence [39, 40] and OI that may result in immune reconstitution inflammatory syndrome (IRIS) [41, 42]. In support of this argument, an RCTs of data from South Africa and Kenya showed that, when given an option, only half of newly diagnosed PLHIV were eligible and ready for same-day initiation [25]. Further studies are needed to investigate the reasons for the major drop in retention during the first 30-days following ART initiation.

Our study dichotomized attrition in terms of LTFU and death. Accordingly, we found that same-day ART initiators had close to 3 times higher risk of LTFU at 6-months compared to > 7 days group. Our findings were similar to “test and treat” program evaluation findings from South Africa, which reported increased odds of LTFU among same-day initiators compared with later initiators (started ART > 1 day) at the end of 6-months follow-up [21]. Additionally, we have observed statistically significant higher LTFU among same-day initiators compared to > 7 days group at 12-months of follow-up. This is consistent with findings from a Nigerian study that showed a significant rate of LTFU at 12-months of ART initiation among PLHIV who started ART within 2 weeks [23]. These findings may point to the need for providing enhanced support and follow-up to PLHIV who started treatment on the initial date of HIV diagnosis. Our study showed no significant difference in the rate of mortality between the two groups at 6- and 12-months periods. This finding was consistent with the systematic review conducted by Ford et al. among PLHIV who started on ART within 2 weeks of HIV diagnosis globally [12].

Despite a suboptimal retention rate and early program losses that have followed the scale-up of universal “test and treat” program especially same-day initiation, evidence showed an increased trend in implementation of rapid ART initiation in LIS [21, 22, 25]. While the push to meet the fast-tracked UNAIDS targets [43] has increased access to early treatment of PLHIV [44], the need for adequate preparation and readiness assessment of PLHIV is becoming evident. We echo the recommendation of Geng and Havlir [45] that the science of rapid ART should pay attention to the “how” part of treatment initiation since the effect of same-day treatment can be altered by patient, provider and system-level factors. More importantly, care should be taken not to force or unnecessarily encourage PLHIV to start treatment on the same-day of HIV diagnosis without ensuring patient comprehension of their result, the need for life-long commitment for treatment adherence and readiness for immediate ART initiation. PLHIV should be properly screened and treated for OI including advanced HIV disease before rapid ART initiation [18] to prevent possible death due to IRIS [41, 42]. Additionally, once initiated, provision of intensified client-centered support for adherence in the initial months of taking ART may be a key initiative to include given the high attrition in the first 30-days of taking ART. Furthermore, in the era of universal “test and treat”, it is important to augment the routine facility-based clinical services with evidence-based initiatives that improve retention such as facility-based adherence clubs [46, 47], community-based adherence support and extra community-based care for high-risk patients [46].

Our study has some limitations that need to be considered while interpreting the study findings. Firstly, given the retrospective nature of the study, we could not address important behavioral factors (e.g. substance use and mental health status) and other provider- and system-level independent factors that may affect the treatment outcomes. Secondly, routine clinical data are usually affected by incomplete information on some of the variables that could affect the analysis. For instance, we have excluded baseline CD4 result from the analysis due to differential missing data. However, this could be an important omission, since a study in Nigeria showed higher risk of LTFU among PLHIV who started ART with higher baseline CD4 cell count. In addition, a significant proportion of PLHIV who started ART under “test and treat” strategy had lower baseline CD4 cell count compared to previously enrolled PLHIV [23]. Since we could not adjust for the effect of this variable, our LTFU result may be overestimated. Thirdly, TO individuals who were referred to another facility within 12-months of ART initiation were excluded from the study due to difficulty to establish their follow-up status at 6^th^ and 12^th^ months. These people may have been less likely to continue their treatment in the other health facility [48]. Furthermore, TO cases were more common among PLHIV who started ART under “test and treat” strategy [23]. This may underestimate attrition due to potential additional loss among TO individuals [48]. Finally, caution should be taken while interpreting the LTFU and mortality results due the difficulty of ascertaining death from LTFU in Ethiopian clinical care settings [49] – some of individuals reported as LTFU could have been dead – leading to overestimation of LTFU and underestimation of death rates. This was also evidenced by a systematic review of studies that traced LTFU patients showing significant deaths among LTFU patients from sub-Saharan Africa ART program [50]. Particularly in Ethiopia, half of LTFU individuals traced through a community survey were found to be dead [49]. Despite these limitations, which are applicable to both groups compared, our study provides useful evidence on relationship between same-day ART initiation and retention outcomes in Ethiopia and builds the evidence-base on this research question in LIS.

## Conclusions

In summary, we found significantly lower retention-in-care among PLHIV who started ART on the same-day of HIV diagnosis at 6- and 12-months follow-up periods compared to those who started their treatment after 7 days. The major drop in retention was in the first 30-days following ART initiation among same-day initiators compared to those who started treatment after 7 days. These findings imply the need for caution when initiating PLHIV on same-day ART while expanding access to treatment to end the public health threat of HIV epidemic by 2030 through rapid ART initiation. Reinforcing adequate preparation and assessment of individuals’ readiness by healthcare workers before initiating PLHIV on same-day ART is vital to maximize retention among PLHIV starting treatment at the first clinical visit. Even with adequate assessment, the major lifestyle change in HIV diagnosis and initiation of a lifelong treatment may warrant enhanced client-centered adherence support and close monitoring of patients during early follow-up periods, particularly in the first 30-days of ART initiation.

## Supplementary Information

**Additional file 1:** **Table 1.** Outcomes of ART initiation at 6- and 12-months ART follow-up by group. **Table 2.** Unadjusted and adjusted RR of study outcomes for same-day ART group.

## Data Availability

The datasets used and/or analyzed during the current study are available from the corresponding author on reasonable request.

## Abbreviations

APHI: Amhara Public Health Institute
ART: Antiretroviral Therapy
ARV: Antiretroviral
AZT: Zidovudine
FDC: Fixed Dose Combination
HIV: Human Immunodeficiency Virus
LIS: Low-income Setting
LTFU: Loss to Follow-up
NNRTI: Non-nucleoside Reverse Transcriptase Inhibitors
OI: Opportunistic Infections
PLHIV: People Living with HIV
RCTs: Randomized Controlled Trails
RD: Risk Difference
RR: Risk Ratio
3TC: Lamivudine
TDF: Tenofovir
TI: Transferred-in
TO: Transferred-out
WHO: World Health Organization
