# Supplementary material for: Effectiveness of same-day antiretroviral therapy initiation in retention outcomes among people living with human immunodeficiency virus in Ethiopia: empirical evidence
Source: BMC Public Health. 2020 Nov 26;20:1802. doi: 10.1186/s12889-020-09887-9 (PMC7690160; doi:10.1186/s12889-020-09887-9)
Supplement: Supplementary file 1 — Additional file 1: Table 1. Outcomes of ART initiation at 6- and 12-months ART follow-up by group. Table 2. Unadjusted and adjusted RR of study outcomes for same-day ART group. [file 12889_2020_9887_MOESM1_ESM.docx]

Supplement Tables

Table 1: Outcomes of ART initiation at 6- and 12-months ART follow-up by group

| **Outcomes** | **No. (%) of participants** | | **Absolute risk difference, % (95% CI)** | **p-value** |
| --- | --- | --- | --- | --- |
|  | **Same-day (n=394)** | **>7 days (n=375)** |  |  |
| Retention at 6-months | 324 (82.2) | 345 (92.3) | 9.8 (5.1, 14.4) | <0.001 |
| Retention at 12-months | 300 (76.1) | 319 (85.1) | 8.9 (3.4, 14.5) | 0.002 |

Table 2: Unadjusted and adjusted RR of study outcomes for same-day ART group

| **Outcomes** | Unadjusted | | | Adjusted** | | |
| --- | --- | --- | --- | --- | --- | --- |
|  | RR* | 95% CI | p-value | RR* | 95% CI | p-value |
| Retention at 6-months^a^ | 0.89 | (0.85, 0.94) | <.001 | 0.86 | (0.81, 0.90) | <0.001 |
| Retention at 12-months^b^ | 0.90 | (0.83, 0.96) | .002 | 0.86 | (0.83, 0.89) | <0.001 |

RR, risk ratio.

*Reference group=persons initiated on ART >7 days after HIV diagnosis.

**Multivariable logistic regression model included the propensity score and other covariates such as:

^a^age, marital status, BMI, IPT, partner’s HIV status and type of ARV regimen.

^b^gender, marital status, IPT, baseline OI and baseline functional status.
